# Nephroprotective Effect of POLYCAN on Acute Renal Failure Induced by Cisplatin in Rats

**DOI:** 10.5402/2012/862104

**Published:** 2012-04-03

**Authors:** Sae-Kwang Ku, Young-Joon Lee, Sung-Dong Lee, Hyung-Rae Cho, Seung-Bae Moon, Ki-Young Kim, Young-Sam Kwon, Joo Wan Kim

**Affiliations:** ^1^Department of Anatomy and Histology, College of Oriental Medicine, Daegu Haany University, 290 Yugok-dong, Gyeongsan, Gyeongsangbuk-do 712-715, Republic of Korea; ^2^The Medical Research Center for Globalization of Herbal Formulation, Daegu Hanny University, 290 Yugok-dong, Gyeongsan, Gyeongsangbuk-do 712-715, Republic of Korea; ^3^Department of Veterinary Internal Medicine, College of Veterinary Medicine, Kyungpook National University, Daegu 702-701, Republic of Korea; ^4^Glucan Corporation and Marine Bio-Industry Development Center, Hoenggye-ri 27 Ilgwang-myeon Gijan-gun, Busan 619-912, Republic of Korea; ^5^Department of Veterinary Surgery, College of Veterinary Medicine, Kyungpook National University, Daegu 702-701, Republic of Korea

## Abstract

We performed to evaluate the effect of POLYCAN (*β*-glucan) on cisplatin-(CDDP-)induced acute renal failure (ARF) in rats. POLYCAN was administered orally once a day for 32 days. Each of 8 rats per group was selected based on the body weight (BW) after acclimatization and they were sacrificed at 5 days after CDDP injection. There was significant (*P* < 0.05) increase of BW after CDDP dosing in all POLYCAN groups than vehicle control and significant (*P* < 0.01 or *P* < 0.05) decrease of absolute and relative kidney weight were detected in all POLYCAN groups compared with vehicle control. In addition, serum BUN and creatinine level in all POLYCAN groups were significantly (*P* < 0.01 or *P* < 0.05) lower than vehicle control and the percentage of degenerative regions significantly (*P* < 0.01) decreased in all POLYCAN groups. As the results of CDDP-induced ARF process, dramatic decrease of the BW, increase of the kidney weight, serum BUN, and creatinine level were detected in vehicle control group compared with sham control group. The changes by CDDP-induced ARF process in POLYCAN groups were significantly and dose-dependently improved compared with vehicle control group. Therefore, POLYCAN has enough potential to develop as a new agent of prevention or treatment for ARF.

## 1. Introduction

Acute renal failure (ARF) is defined as an abrupt renal function decrease enough to result in retention of nitrogenous waste in the body. The hallmark of ARF is progressive azotemia caused by the accumulation of the nitrogenous waste which is accompanied by the wide range of other disturbances depending on the severity and duration of the renal dysfunction. These include metabolic derangement such as metabolic acidosis and hyperkalemia as well as disturbance of body fluid balance and effects on many other organs. ARF is commonly encountered in the medical practice and 4~5% of inpatients in the general medical or surgical ward develop ARF. Abrupt renal failure is a final common pathway for a number of disease processes and is associated with significant morbidity and mortality. Given the high frequency, multiple causes and significant morbidity of the disease, the logical clinical approach to ARF is needed to illuminate the cause and then to proceed to the proper therapy [1, 2].

Cisplatin (*cis*-diaminedichloroplatinum II, CDDP) is extensively used to treat several cancers. Although it is used widely as an antineoplastic agent, the treatment is limited by side effects including nephrotoxicity, emetogenesis, and neurotoxicity [3]. ARF is commonly due to acute tubular necrosis with usually reversible loss of renal function incurred from ischemic or nephrotoxic injuries. ARF by cisplatin in rats exhibits characteristic structural alterations in renal tubular epithelia in association with an impairment of urinary concentrating mechanism. It is typically characterized by polyuria, severe reduction in glomerular filtration rate (GFR), variable decrease in renal blood flow, and the decrease in the urinary concentrating ability [4]. Therefore, CDDP-induced ARF rat model has been regarded one of general animal models to detect the efficacy of drug on the kidney dysfunction. The effect of the drug would be based on the kidney weight, histology, histomorphometric change of kidney, and blood chemistry profiles such as serum BUN and creatinine level [4–6].

Captopril (CAPT) and Losartan (LOSA) are ACE (angiotensin converting enzyme) inhibitors. ACE is an important enzyme for the formation of angiotensin II which causes elevation of blood pressure and constriction of arteries in the body. ACE inhibitors lower blood pressure by inhibiting the formation of angiotensin II. Relaxing the arteries not only lowers blood pressure but also improves cardiac pumping efficiency and cardiac output in patients with heart failure. Since it is a generally accepted fact that CAPT and LOSA have some favorable effects for patients with the renal failure, they have been used as control drugs in nephropathy experiment model [7, 8].

Beta-glucan is a fiber-type polysaccharide derived from the cell wall of yeast, oat, barley fiber, and many mushrooms. The primary use of *β*-glucan is to enhance the immune system [9] and to lower blood cholesterol level [10, 11]. Although some reports showing the evidence of favorable effect of *β*-glucan on the nephropathy [9], the effect of *β*-glucan on the CDDP-induced nephropathy has not yet been reported except for some restrict reports that *β*-glucan inhibited the genotoxicity and general toxicity of CDDP [12]. Antioxidants have been shown to provide protective effects against CDDP-induced nephrotoxicity [13–15]. It is considered that POLYCAN has favorable protective effect on the CDDP-induced ARF because the antioxidative and free radical scavenging activities of *β*-glucans have already been reported [16, 17]. The POLYCAN used in this study is extracted *β*-glucan complex from *Aureobasidium pullulans *SM-2001, UV-induced mutant of *A. pullulans*, it showed somewhat different characteristics from other *β*-glucans derived from other origins [18].

The purpose of this study is to evaluate the effect of POLYCAN (*β*-Glucan, Glucan Corp. Ltd., Korea) originated from *Aureobasidium*, on the CDDP-induced ARF and the efficacy was compared with Captopril and Losartan.

## 2. Materials and Methods

### 2.1. Laboratory Animals

Fifty-six 8-week-old female Sprague-Dawley rats (Samtako Bio, Korea) were used after 13 days of acclimatization. Animals were allocated four per polycarbonate cage in a temperature (20–25°C) and humidity (30–35%) controlled room. Light: dark cycle was 12 hours: 12 hours and feed (Samyang, Korea) and water were supplied freely to access. Forty-eight rats were induced ARF by CDDP injection, and 8 rats were sham control group. All test articles had been administered once a day for 32 days and once intraperitoneally dosed CDDP on 28th day of test article administration (day 28).

### 2.2. Administration and ARF Induction

POLYCAN (Glucan Corp., Korea), and CAPT (sigma, USA) and LOSA (Aurobindo Pharm., India) were used in this study. POLYCAN was stored at 4°C to protect from light and degeneration. POLYCAN was diluted with distilled water and administered by oral gavage using a sonde at each dosage (31.25, 62.5, or 125 mg/kg) once a day for 32 days. CAPT (100 mg/kg in distilled water) and LOSA (20 mg/kg in distilled water) were orally administered as the same manner. To induce ARF, 5 mg/kg of CDDP (Sigma, USA) was once intraperitoneally injected at day 28 (5 days before sacrifice). Sham control group was once intraperitoneally injected with saline only. At the end of the experiment, all animals were sacrifice under anesthesia with Zoletile (Vibrac, France).

All animals were treated according to the Guide for the Care and Use of Laboratory Animals by Institute of Laboratory Animal Resources, commission on Life Science, National Council, USA.

### 2.3. Body and Kidney Weight Change

Body weights were measured at one day before test article administration (day 0), test article administration day (day 1), every 7 days during the test article administration (days 7, 14, 21, and 27), one day after CCDP injection day (day 29), one day before euthanasia (day 32), and euthanasia day (day 33). Animals were fasted overnight (water was not restricted) on days 0, 27, and 32 to reduce the erratum arouse from feeding.

At euthanasia day, the weight of left kidney was measured and the relative weight (%) was calculated divided by BW at euthanasia and times 100 in order to reduce the erratum rose from individual body weight differences.

### 2.4. Measurement of Serum BUN and Creatinine Level

For evaluation of serum BUN and Creatinine level, the whole blood was collected on days 0, 28, and 33 from orbital plexus and serum was separated with general method. Serum BUN and Creatinine levels were measured with an automated blood analyzer (Toshiba 200 FR, Japan) in E-won Clinical Laboratory (Seoul, Korea).

### 2.5. Histopathology and Histomorphometry

Kidney samples were fixed in 10% neutral buffered formalin. After paraffin embedding, 3-4 *μ*m sections were prepared. Representative sections were stained with Hematoxylin and Eosin (H&E) for light microscopic examination.

Percentage of degenerative regions of kidney parenchyma was observed and calculated as percentages of 1 field of kidney (%/200 *μ*m^2^of kidney parenchyma) with an automated image analysis (analySIS Image Processing; SIS, Germany).

### 2.6. Statistical Analysis

All data were calculated as mean ± standard deviation. Statistical analysis was conducted using Mann-Whitney U-Wilcoxon Rank Sum W test (MW test) with SPSS for Windows (Release 6.1.3., SPSS Inc., USA).

## 3. Results

### 3.1. Body Weight Change

Significant decrease of body weight after after CDDP dosing was detected in vehicle control compared with Sham control. However, significant (*P* < 0.05) and dose-dependent increase of body weight after CDDP dosing was detected in all POLYCAN groups. In addition, body weight after CDDP dosing in CAPT and LOSA were also increased compared with vehicle control. However, a significant (*P* < 0.05) decrease of the body weight before CDDP-dosing period was detected in CAPT compared with vehicle control (Table 1).

### 3.2. Kidney Weight Change

Significant (*P* < 0.01) increases of absolute and relative kidney weight were detected in vehicle control compared with Sham control. However, significant (*P* < 0.01 or *P* < 0.05) decreases of absolute and relative kidney weight were detected in all dosing groups compared with Sham control, respectively. Compared with CAPT or LOSA, more favorable decrease of the kidney weight was detected in the lowest dosage of POLYCAN 31.25 mg/kg. In POLYCAN-dosing groups they showed the dose-dependent manner (Table 2).

### 3.3. Change of Serum BUN and Creatinine Level

Changes of serum BUN and creatinine levels before CDDP-dosing period of vehicle control were quite similar to sham control. Although significance (*P* < 0.05) was restricted to the POLYCAN 125 mg/kg and LOSA groups, dramatic decreases of serum BUN and creatinine level were detected in all POLYCAN groups compared with vehicle control, respectively. Changes of serum BUN and creatinine levels after CDDP-dosing of vehicle control significantly (*P* < 0.01) increased than sham control. However, the increased serum BUN and creatinine levels in all POLYCAN groups were significantly (*P* < 0.01 or *P* < 0.05) decreased compared with vehicle control. In POLYCAN groups, the clear dose dependency was observed (Table 3).

### 3.4. Change on Histopathology and Histomorphometry of the Kidney

**Figure 1:**
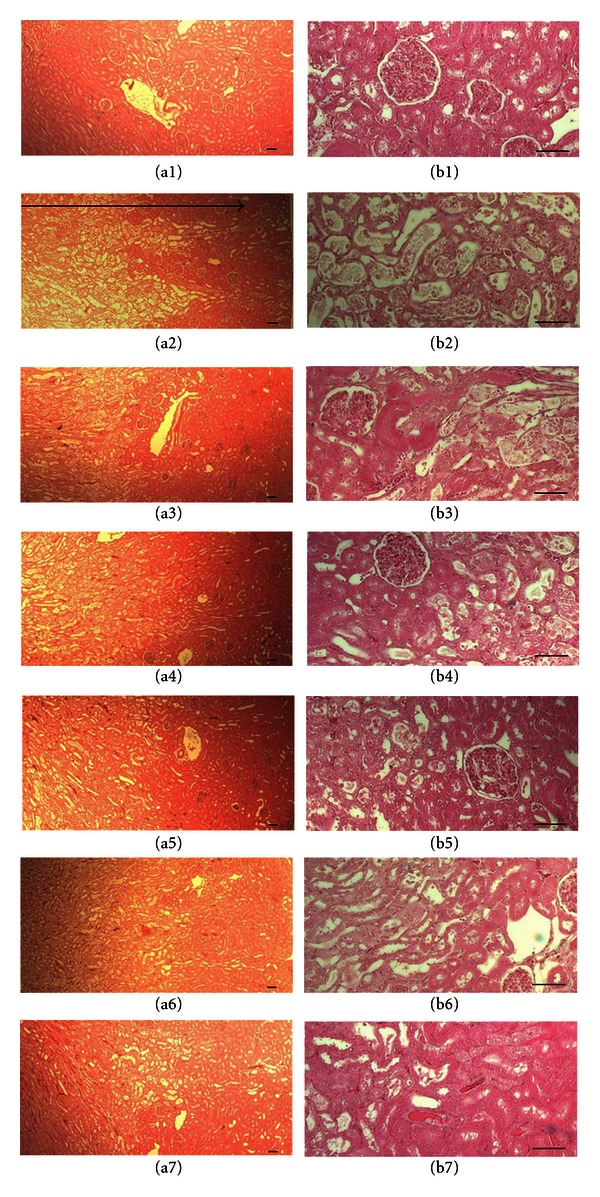
Histological profiles of kidney in Sham (a1, b1), vehicle control (a2, b2), CAPT (a3, b3), LOSA (a4, b4), P 31.25 (a5, b5), P62.5 (a6, b6), and P125 (a7, b7) groups at euthanasia. Note that relatively well-developed histological profiles of kidney were detected in Sham (a1, b1) but epithelial necrosis and desquamation affecting tubules of cortex is detected, and Glomeruli, proximal and distal tubules were severely and locally disrupted in vehicle control (a2, b2). However, these ARF-related histopathological changes on the kidney are dramatically or dose-dependently decreased in all dosing groups tested (a3~a7, b3~b7). Arrow indicated the local disrupted regions. All H&E stain; scale bars = 100 *μ*m.

While classic CDDP-induced ARF histopathological changes which are epithelial necrosis and desquamation in the tubules of cortex and severe and local disruption of glomeruli, proximal and distal tubules were detected in vehicle control, those kinds of ARF-related histopathological changes dramatically and dose-dependently in POLYCAN groups were more decreased than vehicle control (Figure 1). In addition, the percentage of degenerative regions in kidney significantly (*P* < 0.01) increased in vehicle control compared with sham control. However, the percentage of degenerative regions significantly (*P* < 0.01) and dose-dependency decreased in all POLYCAN groups (Table 4).

## 4. Discussion

Decrease of the body weight after CDDP dosing was considered as a result of the direct toxicity of CDDP and or indirect toxicity related to ARF and change of the body weight after CDDP dosing have been used as a valuable index in the efficacy test of ARF [19–21]. Therefore, inhibition of the body weight loss by POLYCAN was considered as a direct or indirect evidence of their efficacy on the CDDP-induced ARF. The decrease of the body weight after CDDP-dosing period in CAPT was considered as the result of high dose (100 mg/kg) of CAPT of which the nontoxic dose in rat is 30 mg/kg at repeated dose for 4 weeks [22].

Since kidney swelling commonly occurs as a result of CDDP-induced ARF, the kidney weight generally increased and it has been provided a valuable index in the efficacy test of ARF [5, 23]. The inhibition of the kidney weight by POLYCAN was considered as a direct evidence of their efficacy on the CDDP-induced ARF.

In CDDP-induced ARF, serum BUN and creatinine levels dramatically increased and changes of serum BUN and creatinine levels have been used as the index in the efficacy test of CDDP-induced ARF [24–26]. Inhibitions of serum BUN and creatinine increases by POLYCAN were considered as the direct evidence of their efficacy on the CDDP-induced ARF.

Since epithelial necrosis and desquamation of renal cortex tubules and severe localized disruptions of glomeruli, proximal, and distal tubules are shown as the general histopathological changes of ARF induced by CDDP in rats, histopathology and histomorphometry have been provided as valuable indexes in the efficacy test on the nephropathy [5, 21, 27]. The inhibitions of the changes on the histopathology and histomorphometry of the kidney by POLYCAN were considered as a direct evidence of their efficacy on the CDDP-induced ARF.

CDDP binds with guanine in DNA and induce apoptosis of cancer. But it can cause renal failure occasionally which seems to be reactive oxygen species action [26].

Although, preventive mechanism of POLYCAN is not known in the CDDP-induced renal failure, it is supposed that POLYCAN inhibit conjugation CDDP with DNA in the kidney [28]. And it is supposed that the ameliorating response of CDDP-induced renal failure because of POLYCAN has anti-inflammatory and immunomodulatory effect dose-dependently [29]. But more study is needed to make clear the mechanism of POLYCAN with a scavenger.

Based on these results, it is considered that POLYCAN has favorable effect on decreasing CDDP-induced ARF process and it has enough potential to develop into a new agent for prevention or treatment of the nephropathy. In addition, more favorable effect was detected in POLYCAN 31.25 mg/kg compared to CAPT 100 mg/kg and LOSA 20 mg/kg. However, the efficacy tests on the various nephropathy models to confirm the potentials of POLYCAN on nephropathy should be performed.

## Figures and Tables

**Table 1:** Change of body weight during the experimental period.

BW	Sham control	Vehicle control	CAPT	P 31.25	P 62.5	P 125	LOSA
Day 0^(1)^	181.63 ± 8.28	182.50 ± 10.42	183.38 ± 6.95	179.75 ± 6.14	180.25 ± 6.25	178.75 ± 6.84	178.25 ± 7327
Day 1^(2)^	164.38 ± 7.19	166.75 ± 7.80	165.50 ± 6.48	162.75 ± 5.85	162.63 ± 5.45	164.00 ± 5.18	162.25 ± 5.60
Day 7	202.13 ± 15.16	206.75 ± 12.43	203.25 ± 6.67	200.50 ± 13.38	197.25 ± 10.25	198.00 ± 10.80	198.38 ± 7.91
Day 14	215.75 ± 16.18	221.75 ± 12.34	216.50 ± 11.11	218.50 ± 14.30	217.13 ± 10.83	211.88 ± 7.08	213.25 ± 9.81
Day 21	233.88 ± 25.16	240.63 ± 14.38	228.38 ± 11.54	232.50 ± 14.85	236.50 ± 15.57	228.88 ± 9.33	226.25 ± 9.47
Day 27	241.13 ± 24.09	249.13 ± 22.97	229.63 ± 15.64	237.88 ± 13.23	239.88 ± 13.04	239.25 ± 8.48	236.50 ± 11.48
Day 28^(3)^	226.75 ± 22.26	234.13 ± 20.36	222.50 ± 11.06	224.38 ± 13.62	227.88 ± 12.80	224.75 ± 6.09	223.50 ± 9.06
Day 32	251.38 ± 24.20	223.50 ± 24.14	222.38 ± 16.66**	230.88 ± 21.96	232.88 ± 15.96	230.88 ± 6.45**	222.00 ± 10.86*
Day 33^(4)^	231.50 ± 25.40	212.88 ± 22.25	209.75 ± 15.25	215.00 ± 20.07	219.63 ± 12.73	217.63 ± 10.95	207.75 ± 8.15**

*n* = 8; (Mean ± S.D., g); ^(1)^One day before of test article administration; ^(2)^first administration of test article after overnight fasted; ^(3)^At CDDP-administration day after overnight fasted; ^(4)^At euthanasia; *P < 0.01 and **P < 0.05 compared to sham by MW test.

**Table 2:** Change of absolute and relative kidney weight at euthanasia.

Kidney weight	Sham control	Vehicle control	CAPT	P 31.25	P 62.5	P 125	LOSA
Absolute weight (g)	0.649 ± 0.092	0.953 ± 0.111*	0.846 ± 0.097*	0.818 ± 0.096^∗,##^	0.795 ± 0.081^∗∗,#^	0.783 ± 0.140^##^	0.848 ± 0.095^∗,##^
Relative weight (%)	0.283 ± 0.045	0.453 ± 0.080*	0.408 ± 0.064*	0.384 ± 0.066*	0.362 ± 0.028^∗,##^	0.361 ± 0.069^∗∗,##^	0.408 ± 0.045*

*n* = 8; (Mean ± S.D.), Relative kidney weight (%) = [(Absolute kidney weight/Body weight at sacrifice) × 100]; *P < 0.01 and **P < 0.05 compared to sham by MW test; ^#^ *P* < 0.01 and ^##^ *P* < 0.05 compared to vehicle control by MW test.

**Table 3:** Change on the serum BUN and creatinine level.

group	Sham control	Vehicle control	CAPT	P 31.25	P 62.5	P 125	LOSA

Serum BUN (mg/dL)							

Day 0^1^	16.91 ± 1.99	16.89 ± 1.53	17.05 ± 2.72	16.83 ± 1.97	17.00 ± 2.30	16.86 ± 1.53	16.88 ± 2.16
Day 28^2^	20.24 ± 1.81	20.10 ± 1.53	18.24 ± 1.18^∗∗,##^	18.33 ± 1.68^∗∗,##^	18.24 ± 1.69**	17.38 ± 1.47^∗,#^	17.25 ± 1.45^∗,#^
Day 33^3^	19.93 ± 1.87	205.98 ± 98.27*	94.53 ± 59.63^∗,#^	72.66 ± 56.77^#^	66.20 ± 50.54^∗∗,#^	63.11 ± 32.39^∗,#^	77.34 ± 48.68^∗,#^

Serum creatinine (mg/dL)							

Day 0^1^	0.55 ± 0.05	0.57 ± 0.03	0.58 ± 0.04	0.56 ± 0.05	0.56 ± 0.03	0.56 ± 0.03	0.57 ± 0.03
Day 28^2^	0.63 ± 0.05	0.65 ± 0.05	0.62 ± 0.02	0.61 ± 0.04^##^	0.59 ± 0.03^##^	0.58 ± 0.03^∗∗,#^	0.60 ± 0.03^##^
Day 33^3^	0.66 ± 0.06	3.76 ± 2.41*	1.65 ± 0.83^∗,#^	1.46 ± 0.72^∗,#^	1.30 ± 0.64^∗,#^	1.19 ± 0.39^∗,#^	1.60 ± 0.98^∗,#^

*n* = 8; (Mean ± S.D.), ^1^first administration day of test article, ^2^at CDDP administration day, ^3^at euthanasia; *P < 0.01 and **P < 0.05 compared to sham by MW test; ^#^ *P* < 0.01 and ^##^ *P* < 0.05 compared to vehicle control by MW test.

**Table 4:** Change on the histomorphometry of kidney at euthanasia.

Histomorphometry^1^	Sham control	Vehicle control	CAPT	P 31.25	P 62.5	P125	LOSA
Degenerative regions	1.73 ± 1.60	78.92 ± 8.56*	56.76 ± 10.36^∗,#^	54.79 ± 10.93^∗,#^	40.50 ± 12.92^∗,#^	33.73 ± 5.77^∗,#^	63.80 ± 10.24^∗,##^

*n* = 8; (Mean ± S.D.); ^1^All histomorphometry was conducted using automated image analyzer as degenerative regions (%/200 *μ*m^2^ of kidney parenchyma); *P < 0.01 compared to sham by MW test; ^#^ *P* < 0.01 and ^##^ *P* < 0.05 compared to vehicle control by MW test.
